# A Spectrum-Aware Priority-Based Link Scheduling Algorithm for Cognitive Radio Body Area Networks

**DOI:** 10.3390/s19071640

**Published:** 2019-04-05

**Authors:** Thien Thi Thanh Le, Sangman Moh

**Affiliations:** 1 School of Computing and Information Technology, Eastern International University, Hoa Phu, Binh Duong City 75114, Vietnam; thanhthien92003@yahoo.com; 2 Department of Computer Engineering, Chosun University, 309 Pilmun-daero, Dong-gu, Gwangju 61452, Korea

**Keywords:** wireless body area network, cognitive radio, cognitive radio body area network, link scheduling

## Abstract

With the development of wireless communication technology, wireless body area networks (WBANs) have become a fundamental support tool in medical applications. In a real hospital scenario, however, the interference between wireless medical devices and WBANs may cause a high packet drop rate and high latency, which is harmful to patients using healthcare services. Nonetheless, cognitive radio is a promising technology for sharing the precious spectrum, which has high efficiency of the wireless resource. Thus, WBANs with cognitive radio capability are also exploited. We propose a spectrum-aware priority-based link scheduling (SPLS) algorithm for cognitive radio body area networks (CRBANs) in a real hospital scenario. In SPLS, three channels are used: DataCh, EDataCh, and CtrlCh for normal data, emergency data, and control messages, respectively. To avoid collision during data transmission, neighboring CRBANs send messages regarding the channel state with CtrlCh before the scheduling. The CRBANs can share DataCh in the time domain for improving the throughput. The SPLS algorithm allows a CRBAN to access idle channels on the licensed and unlicensed spectrum according to the CRBAN traffic. Our simulation results show that the proposed SPLS outperformed the conventional scheme in terms of packet delivery ratio, system throughput, latency, and energy efficiency.

## 1. Introduction

**Figure 1:**
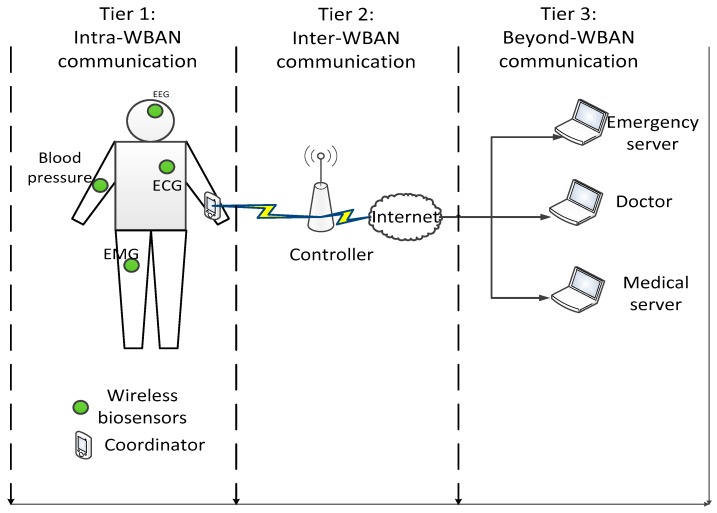
Example of an e-health system.

In the recent years, the development of wireless communication technology has had a great impact on modern medical applications, especially, for healthcare monitoring [1,2]. A medical wireless body area network (MWBAN) comprises multiple wireless biosensor nodes and a coordinator node. The wireless biosensor nodes collect biomedical data such as blood pressure, heartbeat, electrocardiography data, electroencephalogram data, and body temperature which are then transmitted to a coordinator node [1]. An example of an e-health system is shown in Figure 1, in which the collected vital signals at the coordinator node are transmitted to a controller before they are forwarded to the medical server, emergency server or doctor [2,3]. Moreover, the application of MWBANs is also gradually increasing in the advanced healthcare facilities in the hospital [4]. In particular, its medical applications may include patient monitoring or telemedicine.

The traffic priority for various services is summarized in Table 1 according to the IEEE standard 802.15.6 [3]. The IEEE standard 802.15.6 introduced the medium access control (MAC) and physical (PHY) layers of a wireless body area network (WBAN) using unlicensed band ISM and ultra- wideband [3]. However, in crowded places such as hospitals, interference amongst WBANs or between WBANs and other devices using the same radio frequency at the same time can occur. Such interference may result in an unexpected situation that is harmful to the patients. Therefore, the wireless communication systems at a hospital should have low interference in order to ensure the continuity of the signal for e-health systems.

Recently, cognitive radio (CR) has become a paradigm for the efficient reuse of spectrum resources in terms of the opportunistic access of the licensed (primary users) part of the spectrum by unlicensed users (secondary users). Therefore, CR may be a promising solution for the MAC and PHY layers to mitigate the interference with medical WBAN. More specifically, a CR-based-approach has been modeled for WBAN in a hospital environment, which can operate on either an unlicensed or a licensed band [5]. In addition, CR technology has been introduced to reduce the interference in medical environments [6]. The CR technique has been applied to medical WBANs, which aims to improve spectrum usage and mitigate interference in healthcare applications [7].

Taking motivation from the aforementioned works, we propose a link scheduling algorithm for multiple CR body area networks (CRBANs) in the hospital scenario. We assume that each CRBAN collects vital signals which belong to either telemedicine or hospital applications as in [5]. We consider the applications of CRBAN services shown in Table 1 according to the IEEE 802.15.6 standard [3]. The CRBAN with the highest traffic priority or telemedicine applications can be regarded as primary user (PU). On the other hand, the CRBAN with the healthcare monitoring applications or low traffic priority plays the role of secondary user (SU) when the unused licensed spectrum is opportunistically utilized. The interference can be categorized into two types: interference at the medical devices caused by the intra-CRBAN or inter-CRBAN transmission and interference at the CRBAN caused by a neighboring CRBAN transmission or medical devices. However, we only consider the latter case, in which the transmission from a CRBAN is interfered by nearby CRBANs in this paper. The link scheduling algorithm is proposed for channel access by various types of services with different priorities. The CRBAN with the highest traffic priority will occupy the emergency-traffic data channel (EDataCh), while the others will occupy the normal-traffic data channel (DataCh). The control packets of the CRBANs will be transmitted on the control channel (CtrlCh).

The objective of our work is to reduce the interference or maximize the concurrent transmission with a given bandwidth while assuring minimum interference to the medical devices. The link scheduling algorithm has been considered as the most effective algorithm for multiple nodes to access the medium with low interference. The transmission of different WBANs who stay in the same area will be scheduled in time domain. For example, the scheduling scheme allows the WBANs to transmit at different timeslots according to the traffic priority in the IEEE 802.15.6 QoS constraints [8]. In CRBANs, a link scheduling algorithm should allow each CRBAN to access the data channel for transmission while considering the primary users’ (PUs’) activities and medical devices in the hospital. The control channel is used for broadcasting the exchange message amongst the CRBAN, which includes the CRBAN traffic priority. However, multiple CRBANs can reuse a channel through schedule transmission in the time domain. Therefore, the channel utilization increases despite the higher latency in the case of low-priority CRBANs. In the overlay paradigm of cognitive radio, the SUs know the PU’s transmission parameters such as channel gain and transmitted data sequence, and then SUs can start their transmission with the PUs [9]. We also implement overlay CR, in which the CRBANs have knowledge of the transmission schedule of medical devices. As a result, the scheduling algorithm adapts quickly to the medium and ensures its coexistence with medical devices. The simulation results show that the packet delivery ratio drops while the PUs’ activities increase with an acceptable delay. The contribution of the paper could be summarized as follows:

- The usage of cognitive radio has been applied to WBANs in the healthcare applications. The emergency data channel is used only for emergency data of CRBANs while the normal data will be transmitted by using the normal data channel.
- The CRBANs with high traffic priority could play the role of a primary user in the unlicensed channel, which aims to access the spectrum earlier than other CRBANs. As a consequence, the latency of the data with high traffic priority is guaranteed to be less than a threshold value.
- The neighboring CRBANs can share or reuse the spectrum to increase the network throughput because the idle spectrum is limited.

The rest of this paper is organized as follows: in the following section, some related works are reviewed while focusing on CR technology adapted to WBANs for various applications. In Section 3, the system model, including the network model and channel sensing, is introduced. In Section 4, the proposed SPLS algorithm is presented in detail, and the superframe structure, formulation, inter-CRBAN transmission, and intra-CRBAN transmission are discussed with the link scheduling algorithm. In Section 5, the performance of the proposed SPLS algorithm is evaluated via a computer simulation and compared with the conventional scheme. Finally, the paper is concluded in Section 6.

## 2. Related Works

The CRBAN has been introduced for e-health applications in a hospital while considering spectrum sharing on the licensed and unlicensed spectrum [5]. The centralized algorithm is applied to the central controller to allocate the transmission power of the CRBANs on the idle channel in the time domain. The central controller controls the channel access using a request-to-send/clear-to-send (RTS/CTS) algorithm. On the unlicensed band, the RTS/CTS algorithm allows the CRBAN to change the transmission power in order to reduce the interference with medical devices such as life-supporting (LS) devices and non-life-supporting (NLS) devices. However, the controller must update the position of the medical devices as well as PUs and SUs in order to compute the transmission power for the next transmission.

In [6], CRBAN ultra-wideband was investigated and was found to increase the efficiency of spectrum management. Another CRBAN was used in a hospital with the RTS/CTS mechanism with an additional emergency channel for emergency data transmission in [10]. The central controller uses the RTS/CTS mechanism to adapt the transmission power of the CR clients in order to improve the outage probability. In order to categorize the traffic priority, the CR system operates on three channels for normal data, emergency data, and control data. In particular, the control data and normal data channels operate on the 2.4-GHz ISM band while the emergency data channel operates on the 900-MHz ISM band.

In [11], the architecture of CR for medical WBANs was introduced and was divided into three levels: intra-WBAN communication, inter-WBAN networking, and beyond-WBAN networking. The CRBAN is connected to the central control unit for inter-CRBAN networking, and the central control unit is connected to the services such as the hospital, doctors, or emergency services.

The traffic priority of CRBAN is important owing to the type of medical services being used. For example, the data in the CRBAN may be emergency vital data or emotion detection [1,3]. In another work in [12], channel allocation and power adaptation are also applied to the CRBAN to prevent the occurrence of interference while ensuring the quality of services. Another work is focused on the channel security of CRBANs in [13], the CR technology is applied for selecting a secure channel by using cooperative spectrum sensing, which prevents the denial-of-service attacks from an unknown attacker. In [14], CR has been used to implement spectrum sensing based on energy detection with a universal software radio peripheral 2 (USRP2).

In [15], an asynchronous MAC protocol for CRBAN in high interference adapts the transmission power and channel frequency. The coordinator detects the interference in its working channel, and if interference occurs, the coordinator switches to the lowest interference channel. In addition, different power transmission levels are specified according to the interference threshold values. In another work, the MAC protocol for CRBAN is summarized, and the issues and challenges of CR as well as its benefits are explained [16]. In [17], a scheduling algorithm has been developed to assign the transmission of CRBANs in the time domain according to the priority data of the patients. In particular, the transmission of low-priority CRBANs will be scheduled after the superframe length of high-priority CRBANs in time domain, but the scheduling algorithm uses only one data channel without considering emergency data channel.

## 3. System Models

### 3.1. Network Model

We consider network deployment in the hospital with two main e-health applications, telemedicine and hospital information systems, as in [5,10]. The telemedicine application involves providing real-time healthcare service delivery to distant users, such that the sensor nodes send the vital signal to the coordinator, and the coordinator transmits those signals to the gateway or controller [5]. The hospital information system collects the data of patients at the hospital. Because the telemedicine application has a higher priority than the hospital information application, users of telemedicine application are considered as PUs and user of hospital information application is considered as SUs. We assume that PUs will turn on within a specific duration of time according to the needs of patients. In addition, on taking into consideration the traffic priority in the WBAN, we also consider the WBAN with the highest traffic priority in Table 1 as the PU. The notations of the network model and scheduling problem are shown in Table 2.

**Figure 2:**
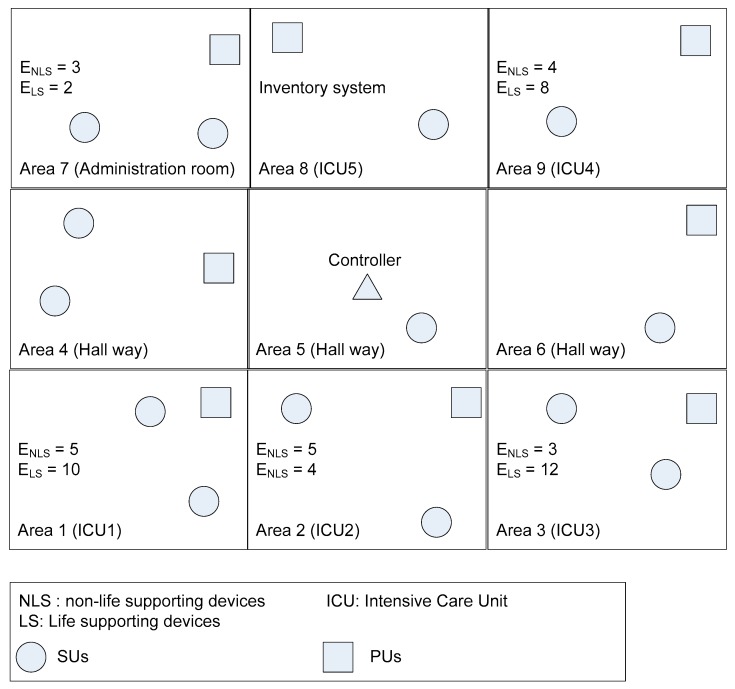
Deployment in the hospital environment.

In order to evaluate the SPLS algorithm, we assume that the three-tier CRBAN is set up as in Figure 1. The particular network scenario of our proposed work in Figure 2 is similar to the hospital scenario in [5,10], which is divided into nine similar rooms, where each room has an area of 9 m^2^. The network consists of a controller and multiple CRBANs with the LS and NLS medical devices. Each CRBAN or SU comprises several sensors on the human body, which is allowed to move freely through the area. In each room, the locations of the NLS and LS medical devices are fixed, and the CRBANs are uniformly distributed in the area. The movement of CR clients is modeled as a random mobility model as in [10].

Taking into consideration the priority of various healthcare services, we define the priority level of CRBANs as follows. The CRBANs can be PUs or SUs depending on the healthcare services as listed in Table 1. In the case of a licensed channel, the CRBANs are SUs with transmissions scheduled according to the CRBAN services. In the case of the unlicensed channel, the nodes for the CRBAN service with the highest priority in Table 1 are regarded as PUs, and the nodes for other CRBAN services are regarded as SUs.

The CRBANs use three kinds of channels called CtrlCh channel, DataCh channel, and EDataCh channel. The CtrlCh is used for transmission of control signals between the coordinators and sensor nodes or between the coordinators and the controller. For example, the CtrlCh is used for the broadcast packet and the beacon packet of the coordinator. The EDataCh is only used for transmission of services with the highest priority shown in Table 1, while the DataCh is used for transmission of normal services. In this system model, CRBANs use the control channel to exchange messages with each other, and the beacon signal of the CRBAN is also transmitted on the control channel when the coordinator senses the idle control channel. The sensor nodes wait for the beacon packet on the control channel and then switch to the data channel for data transmission according to the information in the beacon signal. The CRBAN accesses the control channel by using carrier-sense multiple-access/collision-avoidance (CSMA/CA) protocol. The overlay spectrum is used as an interference mitigation model where the CRBANs use the spectrum that is not occupied by the PUs. The medical devices work on either licensed or unlicensed channels.

The spectrum is divided as follows: the licensed band is specified for wireless medical telemetry service in the spectrum bands 608–614 MHz, 1395–1400 MHz, and 1427–1432 MHz and medical implant communications service in the spectrum band 402–405 MHz; the unlicensed band is the ISM band at 2.4 GHz [5,10]. In [3], as per the IEEE standard 802.15.6, the narrowband band 2400–2483.5 MHz is divided into 79 channels of 1-MHz bandwidth. However, we only consider 20 unlicensed channels at 2.4 GHz for simplicity. In the unlicensed band, the frequency 2483 MHz is selected for CtrlCh because it is not overlapped with the IEEE 802.11 channels, and the frequency 2475 MHz is selected for EDataCh, while DataCh is selected as any idle channel in the ISM band (18 channels). The licensed band is used for the medical devices or PUs, CRBANs sense the vacant channel or the spectrum hole.

We consider two types of transmission: the transmission between the sensor nodes and the coordinator as an intra-CRBAN transmission and the transmission from the coordinator to the controller or the transmission from the coordinator of one CRBAN to the coordinator of other CRBANs as an inter-CRBAN transmission.

In the 2.4–2.5-GHz band, the channel model for intra-CRBAN follows a power law model as per the IEEE standard 802.15.6 as in [18], and the path loss is calculated by:

(1) $$PL(d)=a\log d+b+N_{PL},$$

where *d* is the distance between the transmitter and receiver in mm, *a* (6.60) and *b* (36.1) are parameters of the model, and *N_PL_* is a normally distributed variable with standard deviation σ*_N_* of 3.80.

The inter-CRBAN channel model is considered as a distance-dependent path loss model, the path loss exponent is less than two, and the fading follows a gamma distribution. The mean and variance values follow a power law with respect to the distance between CRBANs, in which the rate of increase of path loss depends on the increase of the distance between two CRBANs [18,19]. In [18,19], the measurement of inter-CRBAN channel model is modeled as the classical distance-dependent path loss model as follows:

(2) $$P_{gain}=ar_{dB}+b,$$

where *P_gain_* is either the mean or variance of the path gain, *r_dB_* = 20log_10_*r*, where *r* is the distance between two CRBANs, and *a* and *b* are the fitting parameters at 2.45 GHz, respectively. The mean of *a* and *b* is −0.05 and −0.19, respectively, and the variance of *a* and *b* is −0.19 and −52.8, respectively.

The path loss between the CRBAN and controller is considered as an indoor path loss model. The transmit power of the medical devices is similar to that in [5,10], and thus the total path loss of a CRBAN in the hospital environment is calculated as follows:

(3) $$PL_{total}=37.7+3.3\log d+16.2n,$$

where *d* is the distance from the medical devices to the CRBAN, and *n* is the number of floors (or walls) the radio signal has to traverse.

The upper bound on the transmit power for NLS and LS devices are defined in [5,8] as follows:

(4) $$P_{NLS}(n)={\left(\frac{D_{NLS}(n)E_{NLS}(n)}{7}\right)}^{2}$$

(5) $$P_{LS}(m)={\left(\frac{D_{LS}(m)E_{LS}(m)}{23}\right)}^{2}$$

where *D_NLS_*(*n*) and *D_LS_*(*m*) are the distances from the CRBAN to the NLS and LS devices, respectively. *E_NLS_*(*n*) and *E_LS_*(*m*) are the electromagnetic interference immunity levels for the NLS and LS medical devices *n* and *m*, respectively, in Figure 2.

Because the NLS and LS devices operate in the same vicinity of CRBANs, the interference probability was defined if the CRBAN causes interference with the medical devices by violating the transmit power constraints *P_NLS_* and *P_LS_*. As in [5,10], the transmit power in the data channel allows a successful transmission from the CRBANs to the controller, which is defined as:

(6) $$Ptx_{data}=\min\{\min_{n}(P_{NLS}(n)),\min_{m}(P_{LS}(m)))\},$$

where *P_NLS_* and *P_LS_* are derived from (4) and (5), respectively.

In [10], in the area with a high electromagnetic interference (EMI) level (mainly due to a large number of life-supporting medical devices), the CRBAN cannot reach the controller with the minimum required signal. In our overlay algorithm, the CRBANs change the operating channel such that they can transmit with a high power while causing no interference with medical devices.

### 3.2. Channel Sensing

The coordinator performs channel sensing on entire licensed channels and unlicensed channels. The coordinator records the signal to interference plus noise ratio (SINR) of each channel in order to determine whether there is any transmission of PUs on a channel. The SINR observed at the coordinator of *CB_i_* in channel *C_k_* is defined as:

(7) $$\gamma_{i}(C_{k})=\frac{P_{r}}{I_{k}+N_{0}},$$

where *P_r_* is the received signal, *I_k_* is the interference power in channel *C_k_*, and *N*_0_ is the additive white Gaussian noise.

In a practical scenario, the value of SINR can be estimated at the PHY layer of the receiver as in [20] using the RSSI (received signal strength indicator) as follows:

(8) $$SINR(t)=10^{(RSSI(t)-\eta_{0}-C-30)/10},$$

where *η*_0_ is the thermal noise, the constant *C* is the measurement offset that is empirically measured in [20] using Chipcon CC2420 on the Telos motes (*C* = 45 dB), and the value of 30 is the conversion of dBm to dB.

As in [21,22,23], channel *C_k_* is idle if there is no PU activity on the licensed channel or no transmission on the unlicensed channel. In such a case, the result of channel sensing at the coordinator of *CB_i_* on channel *C_k_* is denoted as:

(9) $$State_{i}(C_{k},t)=\begin{array}{ccc} Idle & if & \gamma_{i}(C_{k})<\gamma_{th} \end{array},$$

Otherwise:

(10) $$State_{i}(C_{k},t)=\begin{array}{ccc} Busy & if & \gamma_{i}(C_{k})>\gamma_{th} \end{array},$$

**Figure 3:**
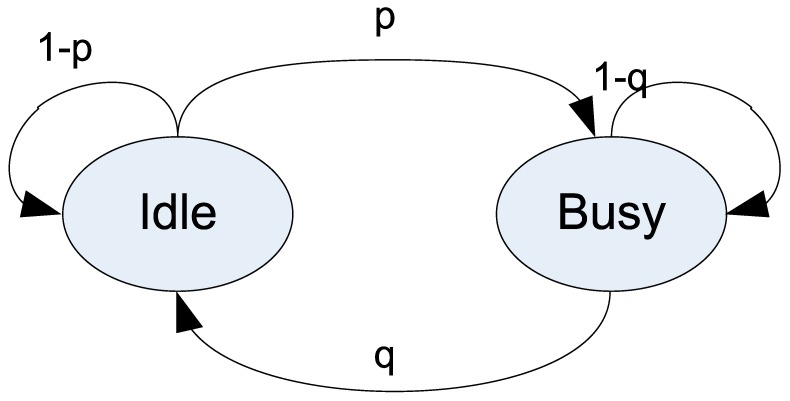
PU activity model.

We assume that the PU user-activity models are similar to those in [21,22,23]. The PU’s user-activity model has two states, which are *Idle* and *Busy*, as shown in Figure 3. The values *p* and *q* are the probability that an idle channel becomes busy and the probability that a busy channel becomes idle, respectively. The durations of the busy and idle times of the PU are defined as *T_busy_*(*k*) and *T_idle_*(*k*), respectively. The arrival of the PU is independent of CRBANs’ activities, and the transition follows a Poisson process in which the lengths of both periods are exponentially distributed with rate λ and mean value *E* = 1/λ. If *q* > *q_thres_* or (1 – *p*) > *p_thres_*, then the coordinator estimates an idle channel *C_k_* at CRBAN *CB_i_* is obtained as:

(11) $$\Delta T_{i,k}=\sum_{t=t_{0}}^{t}\Delta T_{t}(C_{k}),$$

where Δ*T_t_*(*C_k_*) = 1 indicates that *C_k_* is idle during one superframe. The coordinator evaluates the possible time that the duration Δ*T_k_* for which the *CB_i_* can occupy *C_k_* is longer than a threshold duration *T_thres_*, which is equal to the length of a superframe.

The list of idle channels for the data transmission that is observed by the coordinator is denoted as:

(12) $$\begin{array}{c} CI_{i}(t)=\{C_{k}|Status(C_{k},t)=Idle,\\ \Delta T_{k}\geq T_{thres},C_{k}\in C_{L}\cup C_{U}\} \end{array}$$

The coordinator broadcasts the list *CI_i_*(*t*) on CtrlCh to the network to discover the nearby CRBANs. The neighboring discovery and link scheduling steps are explained in the next section.

## 4. Spectrum-Aware Priority-Based Link Scheduling

### 4.1. MAC Superframe Structure

**Figure 4:**
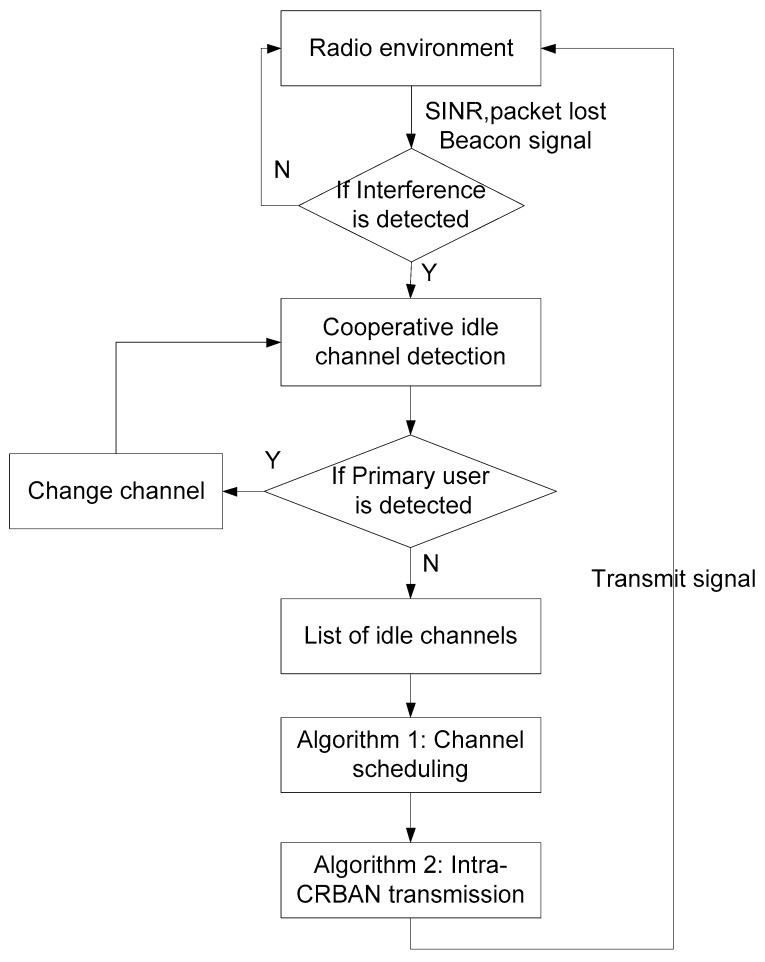
Process of the coordinator at each CRBAN.

**Figure 5:**
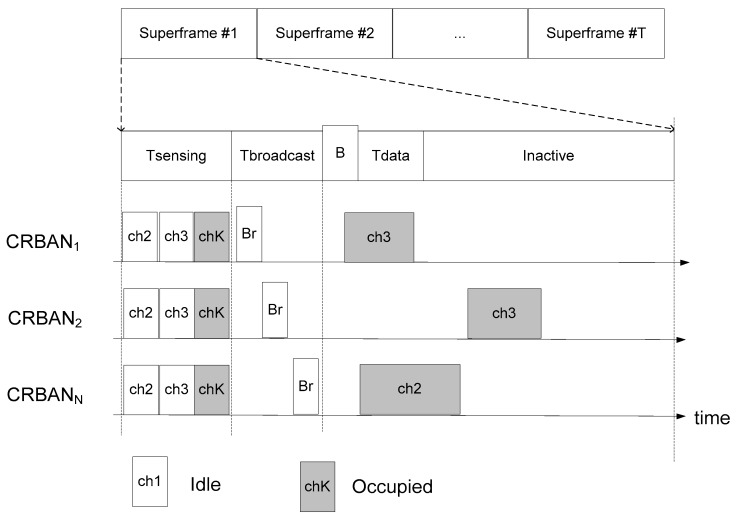
MAC superframe.

The process of link scheduling in CRBANs consists of idle channel detection, channel scheduling, and intra-CRBAN transmission. The framework of the link scheduling for CRBANs is shown in Figure 4, and the MAC superframe is shown in Figure 5. In Figure 4, the coordinator of the CRBAN applies cognitive radio to create the list of channels by performing idle channel sensing as explained in Section 3.2. The coordinator applies the scheduling algorithm to select the channel for data transmission without interfering with the neighboring CRBANs. Each coordinator of the CRBANs broadcasts the beacon signal before starting the intra-CRBAN transmission. However, the sensor nodes wait for the beacon signal and then switch the channel in order to perform the data transmission.

In Figure 5, the superframe is divided into five phases: the sensing, broadcast, beacon transmission, data transmission, and inactive phases. The first phase is the channel sensing phase, which is explained in Section 3.2. In the second phase, the coordinators broadcast the list of idle channels *CI_i_*(*t*) to the network on CtrlCh. Upon receiving the broadcast message from the other CRBANs, each CRBAN selects an idle channel for data transmission. After selecting the idle channel, the coordinator broadcasts the beacon signal to its sensor nodes on CtrlCh. The CRBAN then switches to the data channel to perform the data transmission. In the inactive phase, the coordinator transmits data to the controller, which aggregates the data of the network before forwarding it to different servers. Therefore, the sensor nodes switch to sleep mode during the inactive phase, and the coordinator also switches to sleep mode after forwarding the data to the controller.

### 4.2. Problem Formulation

In this sub-section, we develop the conditions of the scheduling algorithm. The operating time is divided into *T* epochs. Let *C_L_* and *C_U_* be the sets of licensed channels and unlicensed channels, respectively. Then, the set of total channels can be represented as *C* = *C_L_* ∪ *C_U_*. Let us assume that *K* is the set of channels already occupied by the PUs, where *K* ⊂ *C*; the *N* SUs will be scheduled on the set of the remaining channels *A*, where *A* = *C* − *K*. However, the number of SUs is larger than |*A*| channels, and more than one SU will be scheduled in a channel. As there is only one channel for the emergency data, we assume that if a CRBAN has emergency data, the CRBAN will occupy EDataCh by using CSMA/CA. However, as more than one CRBAN can occupy one DataCh, the transmission of the CRBANs is scheduled on DataCh to minimize the interference level. The time duration for the data transmission is defined as the maximum number of timeslots (*t_s_*) that ensures the minimum requirement of latency of the data as follows:

(13) $$T_{data}=t_{s}M\leq Delay_{\min},$$

The length of the superframe for each CRBAN of channel *C_k_* can be calculated as:

(14) $$T_{SF}=T_{S}+T_{brd}+T_{B}+T_{data}+T_{inactive},$$

where the sensing duration is *T_S_* = (*L* + *U*)*t_sense_*, broadcast duration is *T_brd_* = *N* × *t_br_*, the beacon transmission *T_B_*, intra-CRBAN data transmission is *T_data_*, and inactive phase is *T_inactive_*.

We assume that the *i*-th SU (*CB_i_*) takes channel *C_k_* in the superframe *t*, which is defined as *B_i_*(*t*, *C_k_*) = 1; otherwise, *B_i_*(*t*, *C_k_*) = 0. At each CRBAN, the set of neighboring CRBANs selecting the same channel *C_k_* is created as follows:

(15) $$NI_{i}(t,C_{k})=\{CB_{j}|B_{j}(t,C_{k})=1,B_{i}(t,C_{k})=1,d(i,j)<R,1\leq j\leq N\},$$

The list of all neighbors at the *i*-th CRBAN is denoted as:

(16) $$NBI_{i}(t)=\{CB_{j}|B_{j}(t,C_{k})=1,B_{i}(t,C_{k})=1,d(i,j)<R,1\leq j\leq N,C_{k}\in CI_{i}(t)\},$$

The coordinator of the CRBAN calculates the interference level as the average number of SUs operating in the same channels in the previous transmission as follows:

(17) $$W_{i,k}=\sum_{1\leq j\leq M}^{}B_{j}(t-1,C_{k}),$$

To prevent the occurrence of interference, two neighboring SUs cannot take the same channel at the same time *t*. That is:

(18) $$B_{i}(t,C_{k})+\sum_{j\in NI_{i}(t,C_{k})}^{}B_{j}(t,C_{k})\leq 1,$$

However, the list of idle channels at each CRBAN is limited and depends on the number of PUs. Thus, we assume that *X* neighboring CRBANs share the same channel *C_k_* in a time division multiple access (TDMA) manner in which the data transmission for *X* CRBANs is *T_NBX_*. The *X* neighboring SUs can form a group called *NB*2 as follows:

(19) $$NB2=\{CB_{i}\cup CB_{j}|CB_{i}\in NBI_{j},CB_{j}\in NBI_{i},C_{k}\in \{CI_{i}(t)\cap CI_{j}(t)\},$$

The CRBANs in *NB*2 can be scheduled on the same channel *C_k_* in a TDMA manner with the length of transmission *T_NB_*_2_ as follows:

(20) $$T_{NB2}=\sum_{x\in NB2}\sum_{t_{x}=1}^{X}T_{x}B_{x}(t_{x},C_{k})\leq T_{\max},$$

In the scheduling problem, we aim to maximize the number of SUs over (*C*–*K*) channels, where *A* = *C* − *K*:

(21) $$\max\sum_{k=1}^{A}\sum_{i=1}^{N}B_{i}(t,C_{k}),$$

subject to:

(22) $$\sum_{k=1}^{A}B_{i}(t,C_{k})\leq 1,$$

(23) $$1<B_{i}(t,C_{k})+\sum_{j\notin NBI_{i}(t,C_{k})}B_{j}(t,C_{k})\leq SF_{i}$$

(24) $$T_{NB2}=\sum_{i\in NB2}\sum_{t_{i}=1}^{X}T_{i}B_{i}(t_{i},C_{k})\leq T_{\max}$$

The constraint of formulation (21) can be explained as follows. Equation (22) implies that each SU requires one data channel for data transmission. Two non-neighboring SUs can transmit at the same time on the same channel for which the length of transmission is equal to one superframe as in (23). In (24), the total transmission of SUs on one channel cannot exceed a threshold *T_max_* in order to ensure maximum latency, where *T_i_* is the transmission length of one SU, and *X* is the number of neighboring CRBANs that can share one channel. *B_i_*(*t_i_*, *C_k_*) denotes that *CB_i_* will start intra-CRBAN transmission at the start time *t_i_* on channel *C_k_*.

### 4.3. Inter-CRBAN Transmission

The inter-CRBAN transmission can be explained as follows. After the spectrum-sensing phase represented in Figure 3, each CRBAN creates an idle channel list. Let us assume that at the initial phase, each SU does not know its neighbors. Therefore, they may take the same idle channel, which leads to a collision. To avoid this situation, the SUs broadcast a message and then receive the message from other SUs in order to create the list of neighbors. Owing to its limited transmission range, the CRBAN will create a list of neighbors based on the successfully received messages.

**Figure 6:**
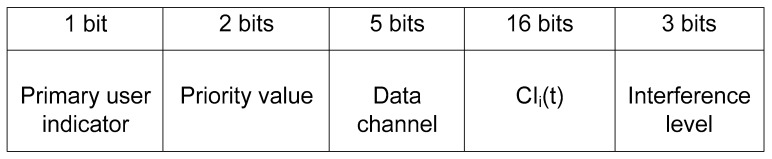
Broadcasting message for CRBANs.

The exchanged messages are broadcasted on CtrlCh, which contains the primary indicator, priority value, data channel, list of idle channels, and the interference value in 2 bytes, as shown in Figure 6. The PU indicator is either 0 or 1. The priority value of the CRBAN is as shown in Table 1. The filed “data channel” is divided into two cases: for emergency CRBANs, the “data channel” is EDataCh, while in the other case, it will be filled after the scheduling algorithm. *CI_i_*(*t*) is the list of idle channels at the *i*-th CRBAN. The interference value is calculated as in (17) using the historical values of the average number of SUs that share the same data channel.

### 4.4. Link Scheduling Algorithm for CRBANs

The problem formulation (21) can be solved using a scheduling algorithm. After the inter-CRBAN transmission or broadcasting steps shown in Figure 4, if two neighboring CRBANs take the same data channel *C_x_*; the CRBAN with the highest priority value will transmit on channel *C_x_* immediately with transmission time *T_i_*, while the other CRBAN will wait for *T_i_* before it starts transmission. The scheduling algorithm at the coordinator on DataCh is shown in Algorithm 1. The coordinator of *CB_i_* receives the broadcasting message from its neighbors in line 2. Then, the coordinator of *CB_i_* updates the list of idle channels of its neighbors in lines 3 to 6. In Algorithm 1, *CB_i_* considers two cases, which are (1) if *CI_i_*(*t*) of *CB_i_* and that of the neighbors are overlapped, which is shown in lines 7 to 22; (2) if none of the neighbors of *CB_i_* sense the same idle channel as *CI_i_*(*t*), which is shown in lines 23 to 26.

**Figure 7:**
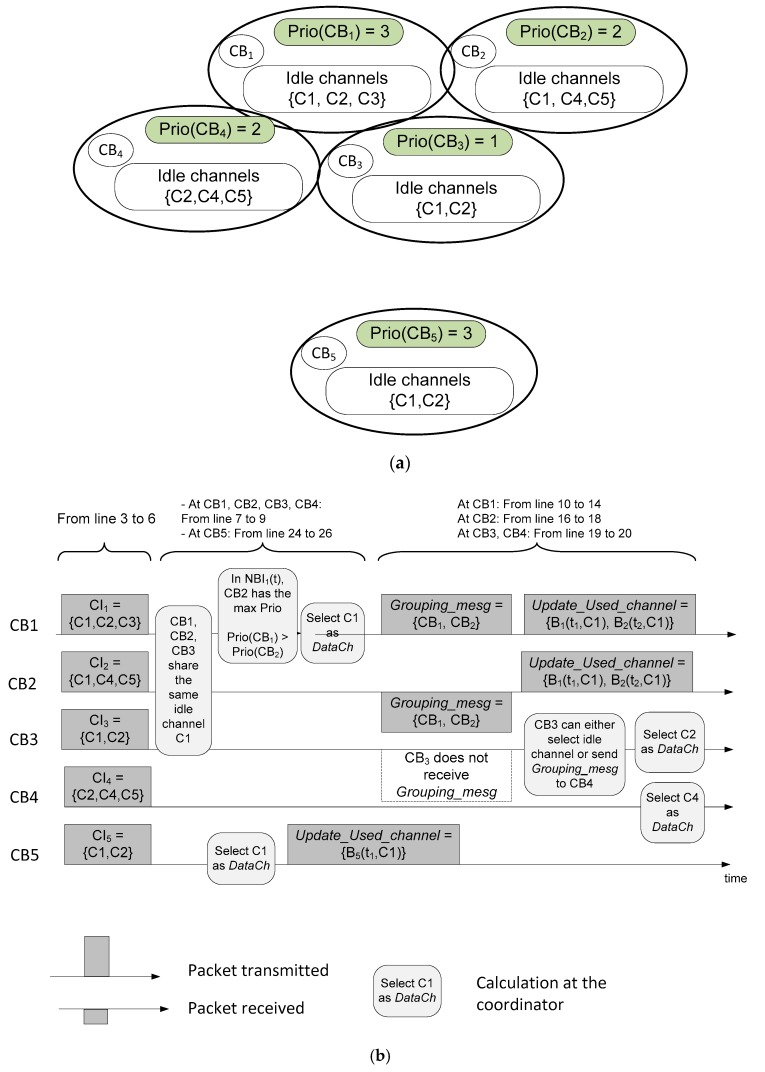
An example of the scheduling algorithm: (**a**) network scenario; (**b**) sending and receiving steps at CRBANs.

In order to illustrate the Algorithm 1, an example is shown in Figure 7. In Figure 7, the network has five CRBANs denoted as *CB*_1_, *CB*_2_, *CB*_3_, *CB*_4_, and *CB*_5_; each CRBAN has the priority value and the list of idle channels. We assume that *CB*_1_, *CB*_2_, *CB*_3_, and *CB*_4_ are neighbors with different priority values. In Figure 7, if *CB*_1_, *CB*_2_, and *CB*_3_ share the same idle channel C1, the priority value of *CB*_1_ is larger than that of *CB*_2_ and *CB*_3_. Assuming that only two CRBANs share the same data channel, *CB*_1_ will select C1 as a data channel and then broadcast a *Grouping_message* to *CB*_2_. Therefore, *CB*_1_ and *CB*_2_ will update the data channel. However, *CB*_3_ does not receive the grouping message, and *CB*_3_ can either select a data channel or send grouping message to *CB*_4_. Because *CB*_5_ does not have any neighbor, *CB*_5_ can select any data channel in its sensed idle channels.

However, in the case of two CRBANs having the same priority (same emergency data) in the same area, the available emergency data channel is only one channel, and one CRBAN has to select another data channel for transmission based on the interference level. If two CRBANs are scheduled in EDataCh in the time domain, one CRBAN is delayed by a period of time Δ*T*, which is equivalent to the transmission time of other CRBAN. If Δ*T* is less than the acceptable latency, two CRBANs can be scheduled in EDataCh.

In [5,10], the control channel is used for transmitting the RTS/CTS between the SUs and controller; nonetheless, the control channel in our work is used for transmission of the exchange message amongst the SU and the beacon signal. Therefore, the SUs have knowledge of the usage of idle channels in the network. The data transmission from the SUs to the controller occurs on the data channel with an acceptable delay, which is considered in the exchange messages. Therefore, the SUs reuse the data channel without interfering with the PUs or medical devices.

**Algorithm 1** Link scheduling algorithm at the coordinator on DataCh.**Input:***N* CRBANs, at each CRBAN {List of idle channels *CI_i_*(*t*), list of neighbors *NBI_i_*(*t*, *C_k_*), maximum transmit power *PTx_data_*}**Output:** A set of valid data channels {*C_x_*} for *N* CRBANs1. **While** all WBANs are not scheduled2.  At coordinator of *CB_i_*: received the *NBI_i_*(*t*) (list of neighbors)3.  **For** each *CB_y_*∈*NBI_i_*(*t*)4.   Check the list of idle channels *CI_y_*(*t*) of each *CB_y_*5.   *CI_NBI_*(*t*) = *CI_NBI_*(*t*) ∪*CI_y_*(*t*)6.  **End for**7.  **If** (*CI_NBI_*(*t*) == *CI_i_*(*t*)) 8.   Find *CB_x_* with the highest priority value in *Prio*(*CB_x_*) = max(*Prio*(*NBI_i_*(*t*))9.   **If** (*Prio*(*CB_i_*) > (*Prio*(*CB_x_*))10.    Broadcast *Grouping_Mesg* = {*CB_x_*, *CB_i_*} to *NBI_i_*(*t*)11.    *CB_i_*, *CB_x_* takes the first channel in *C_z_* ∈ *CI_i_*(*t*),12.    Set *T_NB_*_2_ as (24)13.    Set *B_i_*(*t_i_*, *C_z_*) = 1; *B_x_*(*t_x_*,*C_z_*) = 1;14.    Broadcast the *Update_Used_Channel* = {*B_i_*(*t*, *C_z_*), *B_x_*(*t_x_*, *C_z_*)}15.   **Else**16.    Waiting for the *Grouping_Mesg*17.    **If** (*CB_i_*∈*Grouping_Mesg*)18.     Update *B_i_*(*t_i_*, *C_z_*) = 119.    **Else**20.     *CB_i_* takes the random channel *C_z_* ∈ *CI_i_*(*t*)21.    **End if**22.   **End if**23.  **Else**24.   *CB_i_* will select a channel *C_z_* = {*C_k_*|*C_k_* ∈ *CI_i_*(*t*), max(Δ*T_t_*(*C_k_*))} as in (17b)25.    Broadcast the *Update_Used_Channel* = {*B_i_*(*t*, *C_z_*)}26.  **End if**27.  *Update_Used_Channel* = set of valid data channels {*C_x_*} for *N* CRBANs28. **End while**

### 4.5. Intra-CRBAN Transmission

**Figure 8:**
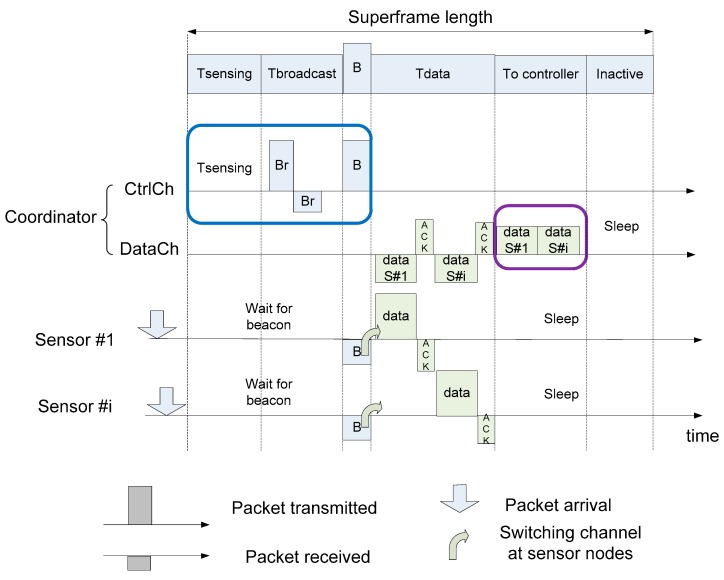
An example of intra-CRBAN transmission.

The intra-CRBAN transmission is explained in Algorithms 2 and 3, and Figure 8. For the intra-CRBAN transmission, if the coordinator does not receive the packet of the sensor nodes after a threshold, the coordinator assumes that interference occurs or a PU arrives. Each sensor node has different traffic types such as emergency traffic or normal traffic.

**Algorithm 2** Intra-CRBAN transmission at the coordinator.**Input:** set of {*M*} sensor nodes, historical packet delivery ratio *PDR*{*t* − 1,*M*}**Output:** A TDMA scheduled for *M* sensor nodes1. Estimate the number of packet delivery ratio at each sensor *PDR*(*t* − 1) = {*PDR_j_*|*j* ∈ M}2. Sort *PDR* in descending order3. **For** each sensor nodes4.  Schedule the *j*-th sensor with the lowest *PDR*(*j*) at the first available timeslot5.  Remove *j* of {*M*}6. **End for**7. Broadcast beacon signal in CtrlCh, Beacon = {schedule({*M*}), index of DataCh *C_k_*, length of superframe *T_i_*, start time of next superframe}

**Algorithm 3** Intra-CRBAN transmission at the sensor nodes.**Input:** set of {*M*} sensor nodes, historical packet delivery ratio *PDR*{*t* − 1,*M*}**Output:** A TDMA scheduled for *M* sensor nodes1. Wait for beacon signal on CtrlCh2. Change the working channel to DataCh according to beacon signal3. Transmit data in the assigned timeslot4. Go back to sleep after receiving ACK from the coordinator

In a CRBAN, the coordinator calculates and records the packet delivery ratio (PDR) of the sensor nodes in the consecutive superframes, and then sorts the PDR values in the descending order as shown in lines 1 and 2 in Algorithm 2. The coordinator assigns the timeslot for each sensor node according to the PDR as shown in Algorithm 2 from lines 3 to 5. After scheduling the sensor nodes into the TDMA superframe, the coordinator broadcasts the beacon signal to the sensor nodes as shown in line 7. However, the sensor nodes only wait for the beacon signal (line 1 in Algorithm 3) and change to the working channel to transmit the data packet (from lines 2 to 4). An example of Algorithms 2 and 3 is illustrated in Figure 8 for a full superframe length. In Figure 8, after the coordinator receives the data packets from its sensor nodes, the coordinator forwards the aggregated data to the controller and then goes back to sleep in DataCh. In the inactive time, the other CRBAN that takes the same DataCh is active and starts the intra-CRBAN transmission.

## 5. Performance Evaluation

In this section, we evaluate the performance of the proposed algorithm using the MATLAB simulator. We consider the network area as in Section 3.1, which is similar to the network in [10]. We also compare our work with the RTS/CTS protocol in [10]. In [10], a CRBAN sends its RTS message to the central controller. Then, the central controller allows the CRBAN to transmit data in the data channel with the required transmit power. We select the dual CR WBAN for our comparison work because the hospital application is used in [10], and the traffic priority is considered by using an emergency data channel. In [10], WBANs are applied the cognitive radio, and sensor nodes transmit vital signals to the controller by using the RTS/CTS protocol.

### 5.1. Simulation Environment

**Figure 9:**
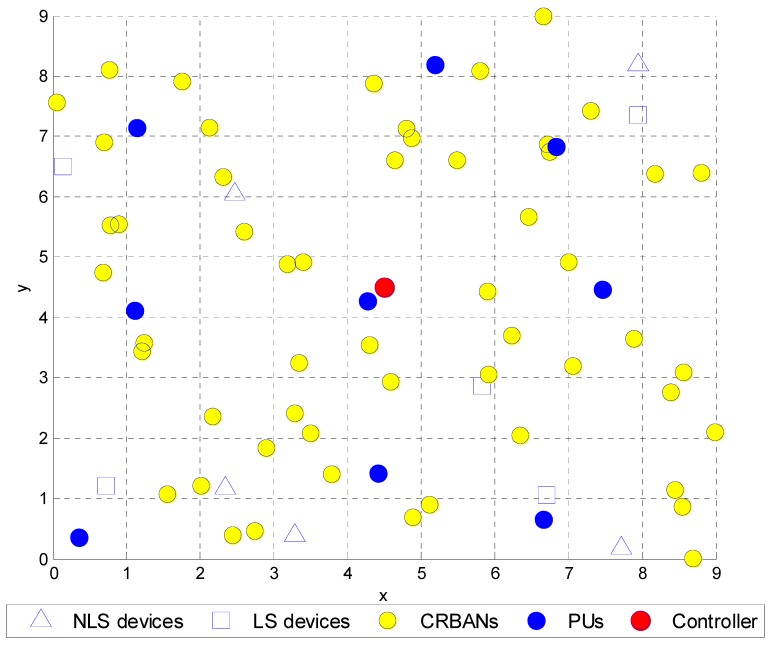
Network deployment.

The network area is shown in Figure 9 and is divided into nine similar rooms, and each room has an area of 9 m^2^. In the simulation scenario, each room has one LS device and one NLS device. The ENLS and ELS of each room, which are the EMI immunity levels for the NLS device n and LS device m, are shown in Figure 2. The transmission range from the CRC to the CRBANs is 10 m to cover the whole network area. The transmission range of each WBAN is 2 m, in which the sensor nodes transmit and receive signals to/from the coordinator. In this simulation, we consider the performance on an unlicensed channel at 2.4 GHz, which is divided into 10 channels with a 1-MHz bandwidth for simplicity (2400–2483.5 MHz). We study four performance metrics: packet delivery ratio, packet delay, throughput, and energy consumption. To consider the impact of the PUs’ activities at each superframe, we vary the number of PUs in each area, in which the probability that the PUs are on is 0.5 (*p_thres_* = 0.5 and *q_thres_* = 0.5 as in Section 3.1). We assume that the probability of misdetection is 0.01. For simplicity, we only implement sensors that generate a normal data packet with various packet generation rates. We also vary the number of CRBANs in each area. However, the NLS and LS devices are on during the simulation time. The interference is considered to occur between CRBANs and medical devices or between the PUs and SUs. In the former case, the interference occurs when the transmit power is higher than the acceptable level or when the status of the medical devices is incorrectly reported at the CRC. In the latter case, the interference occurs when the SUs and PUs transmit at the same time. The simulation parameters are listed in Table 3.

### 5.2. Simulation Results and Discussion

#### 5.2.1. Packet Delivery Ratio

**Figure 10:**
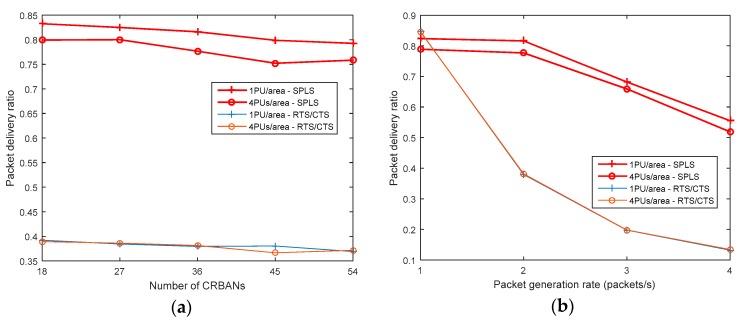
Packet delivery ratio: (**a**) varying the number of CRBANs; (**b**) varying packet generation rate.

The packet delivery ratio (PDR) is considered as the ratio of the number of successfully received packets to the number of sent packets at the CRBAN. The PDR result is shown in Figure 10. The PDR depends on the packet generation rate and number of CRBANs in the network. However, the PDR decreases slightly in the scenario with a high number of PUs while increasing the number of CRBANs and number of PUs, as shown in Figure 10. If the packet generation rate increases, the PDRs of both algorithms decrease as shown in Figure 10. In the multiple CRBANs network, the coordinator of the CRBAN starts its neighbor discovery and exchanges information with the specific neighbors in order to share the same channel in the presence of the PU. Therefore, the CRBAN transmission is scheduled into an idle channel, which ensures the successful transmission. However, when the packet generation rate at the sensor nodes increases, the sensor can transmit packets within the scheduled superframe in the time domain. Therefore, some packets at the sensor nodes may be dropped because of collision.

#### 5.2.2. Packet Delay

**Figure 11:**
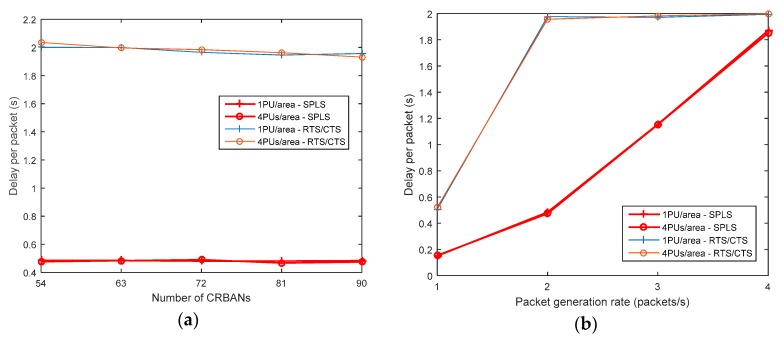
Delay per packet: (**a**) Varying the number of CRBANs; (**b**) Varying packet generation rate.

The packet delay is considered as the time from which a packet is received at the CRC to the generated time at the CRBAN. The packet delay is shown in Figure 11. As with the PDR, the packet delay also depends on the packet arrival rate and number of PUs. In Figure 11, the latency per packet of the SPLS is lower than that of the RTS/CTS. In the SPLS, the sensor nodes transmit the data packet according to the assigned schedule in an idle channel. However, in the RTS/CTS algorithm, the coordinator allows the sensor nodes to send the data packet when the coordinator senses an idle channel. However, the packet delay of the SPLS is increased when the number of PUs and CRBANs is increased. Figure 11 shows the delay when the number of CRBANs is increased while the packet generation rate is 2 packets/s. In Figure 11, the delay of the SPLS is high when the packet generation rate increases in the case of 72 CRBANs in the network.

#### 5.2.3. Network Throughput

**Figure 12:**
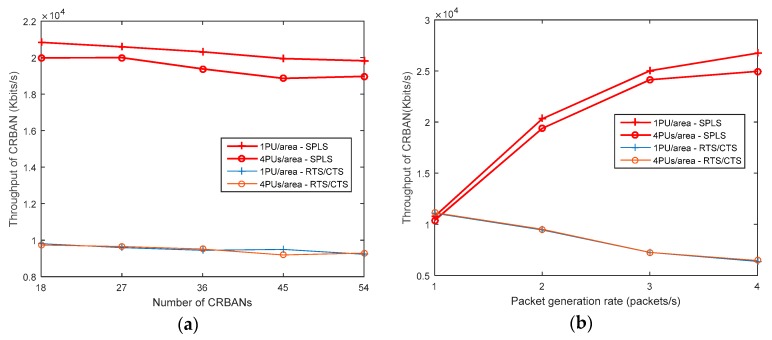
Throughput of CRBAN: (**a**) varying the number of CRBANs; (**b**) varying packet generation rate.

The network throughput is considered as the number of successful packets in the simulation time, which is measured in Kbits/s. The network throughput is shown in Figure 12, wherein the number of CRBANs and the packet generation rate are varied. In Figure 12, the throughput of each CRBAN decreases slightly because the network density increases with the decrease in the PDR, as shown in Figure 10. However, the throughput of each CRBAN increases with the packet generation rate in the scenario of 72 CRBANs. The SPLS realizes a superior performance than the RTS/CTS. In Figure 12, the throughput of the CRBANs increases with the increase in the packet generation rate, and the sensor nodes can successfully transmit more packets according to the schedule in the time domain. However, the network throughput depends on the number of PUs in the network, and the network throughput in the case of 4 PUs per area is lower than that in case of 1 PU per area.

#### 5.2.4. Energy Consumption

**Figure 13:**
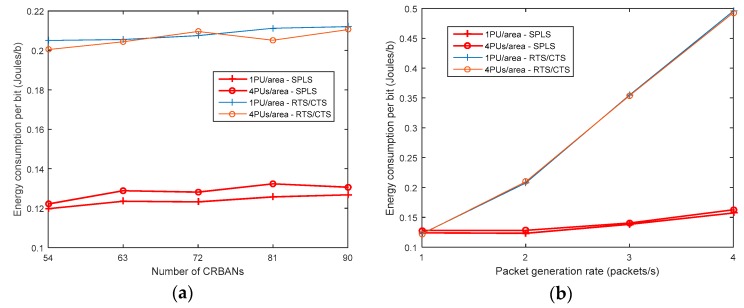
Energy consumption per bit: (**a**) Varying the number of CRBANs; (**b**) Varying packet generation rate.

The energy consumption is considered to be the energy required for transmitting and receiving data packets and negotiation packets, the energy for sensing channels, and the energy for switching channels. We assume that the energy consumed during the transmission and receiving of packets is similar to that in [15]. The energy consumption per bit is calculated as the ratio of the total energy consumed during the transmission over the number of successful received packets in bits [24]. The total energy consumption for transmitting and receiving data packets increases with the number of CRBANs and the packet generation rate. The energy consumption is shown in Figure 13, which shows that the SPLS consumes less energy than the RTS/CTS algorithm. The energy consumption of both the algorithms is increased as shown in Figure 13. The presence of PUs results in a high energy consumption of the CRBAN when varying the network density. In contrast, the energy consumption is increases gradually in Figure 13 according to the packet generation rate. In Figure 13, the energy consumption per bit at the packet generation rate of 1 and 2 packets per second is similar. This is because, as the packet generation rate increases from 1 to 2 packets per second, the total energy consumed during transmission is increased but the number of successful received packets is also increased as shown in the Figure 10. As a result, the energy consumption per bit is not increased in this case. When the packet generation rate increases up to 3 and 4 packets per second, PDR gradually decreases as shown in Figure 10, resulting in the increased energy consumption per bit in Figure 13. In the SPLS, the energy is consumed in the sensing duration only one time per superframe before negotiating with the neighbors. The CBRANs negotiate with the neighbors before creating a cluster of two CRBANs sharing the same idle channel. As a consequence, the CRBANs do not sense the channel as in the case of the RTS/CTS.

## 6. Conclusions

In this paper, the spectrum-aware link scheduling algorithm for CRBANs in the multi-channel environment has been proposed. In order to guarantee that CRBANs can be adapted to a high-interference scenario with an acceptable performance, the use of an SPLS algorithm is required in CRBANs. The proposed SPLS algorithm allows a CRBAN to switch to an idle channel with low latency. The traffic of the CRBANs is also taken into consideration by using a separate channel for emergency data in order to improve the network reliability. The coordinator performs the channel sensing and negotiation with the neighbors in order to reduce the energy consumption. In addition, the CRBANs negotiate for the schedule, which results in reduced collisions owing to the use of the control channel. The scheduling algorithm allows two neighboring CRBANs to share the same idle channel, which increases the probability of successful transmission in the presence of the PU’s activity. The simulation results show that the proposed SPLS realizes a superior network performance with lower energy consumption as compared to the conventional scheme. In future works, we intend to improve the present work in terms of energy efficiency and high reliability.

## Figures and Tables

**Table 1:** WBAN priority for various services [3].

Priority	WBAN Services
0 (lowest)	Non-medical services
1	Mixed medical and non-medical services
2	General health services
3 (highest)	Highest priority medical services

**Table 2:** Notations for spectrum-aware priority-based link scheduling algorithm.

Notations	Explanation
*N*	Number of CRBANs
*CB_i_*	*i*-th CRBAN, 1 ≤ *i* ≤ *N*
*M*	Number of sensors per CRBAN
*s_ij_*	*j*-th sensor of *i*-th CRBAN, 1 ≤ *j* ≤ *M*
*t_s_*	Timeslot
CtrlCh	Common control channel
EDataCh	Emergency data channel
DataCh	Normal data channel
*q_thres_*	Threshold value of probability that a busy channel becomes idle
*p_thres_*	Threshold value of probability that an idle channel becomes busy
*d*(*i*,*j*)	Distance between the coordinators of two CRBANs
*R*	Transmission range of a CRBAN
*U_k_*	*k*-th unlicensed channel *U_k_* ∈ *C_U_*, 1 ≤ *k* ≤ *U*, where *U* is the number of unlicensed channels, and *C_U_* is the set of unlicensed channels
*L_k_*	*k*-th licensed channel *L_k_* ∈ *C_L_*, 1 ≤ *k* ≤ *L*, where *L* is the number of licensed channels, and *C_L_* is the set of licensed channels
*K*	Number of channels occupied by PUs
*A*	Number of remaining channels to schedule *N* CRBANs
γ_i_(*C_k_*)	Observed SINR at *i*-th CRBAN in a channel *C_k_*, *C_k_* ∈ *C_L_* ∪ *C_U_*
γ_*th*_	SINR threshold
*CI_i_*(*t*)	List of idle channels observed at *i*-th CRBAN
*B_i_*(*t*,*C_k_*)	*B_i_*(*t*, *C_k_*) = 1 means that *i*-th CRBAN occupies channel *C_k_* in superframe *t*; *C_k_* ∈ *CI_i_*(*t*).
*NI_i_*(*t*, *C_k_*)	List of neighbors that sense the same *C_k_* as *i*-th CRBAN
*NBI_i_*(*t*)	List of neighbors of *i*-th CRBAN
Statei(*C_k_*, *t*) = {Idle, Busy}	Status of channel *C_k_* of *CB_i_* at time *t*
*SF_i_*	Length of superframe of *i*-th CRBAN
SF_max_	Maximum length of superframe in the schedule
*IF_i_*(*t*, *C_k_*)	Interference level of *i*-th CRBAN at channel *C_k_*
*T_max_*	Maximum number of superframe in the schedule

**Table 3:** Simulation parameters.

Parameter	Value
Bit rate	250 Kbps
Synchronization time with CRC	10 ms
Data slot time	10 ms
Exchange message length	2 bytes
Number of CRBANs	54–90 (default: 72) or 5~10 per area (default: 7)
Number of sensor per CRBAN	10
Number of PUs	9–36 (1–4 PUs per area)
Packet generation rate	1–4 packets/s at each sensor (default = 2 packets/s)
Number of unlicensed channels	10
Superframe length	100 ms
Frequency	2400–2483.5 MHz
Bandwidth	1 MHz
Data packet size	50 bytes (normal data)
Transmitted power of CRBAN	10 dBm
Transmission range of controller	10 m
Transmission range of CRBAN	2 m
Transmit current [15]	17.4 mA
Receive current [15]	19.7 mA
Energy consumption per channel switching [15]	2 mJ
Voltage	3.3 V
Receiver sensitivity at controller	−80 dBm

## References

[1] Ullah S., Khan P., Ullah N., Higgins H., Saleem S., Kwak K.S. (2009). A Review of WBANs for Medical Applications. Int. J. Commun. Netw. Syst. Sci..

[2] Movassaghi S., Abolhasan M., Lipman J., Smith D., Jamalipour A. (2014). Wireless Body Area Networks: A Survey. IEEE Commun. Surv. Tutor..

[3] IEEE (2012). IEEE Standard for Local and Metropolitan Area Networks—Part 15.6: Wireless Body Area Networks.

[4] Naeem M., Pareek U., Lee D.C., Khwaja A.S., Anpalagan A. (2015). Wireless Resource Allocation in Next Generation Healthcare Facilities. IEEE Sens. J..

[5] Phunchongharn P., Hossain E., Niyato D., Camorlinga S. (2010). A Cognitive Radio System for E-health Applications in a Hospital Environment. IEEE Wirel. Commun..

[6] Chavez-Santiago R., Nolan K., Holland O., De Nardis L., Ferro J., Barroca N., Borges L., Velez F., Goncalves V., Balasingham I. (2012). Cognitive Radio for Medical Body Area Networks using Ultra Wideband. IEEE Wirel. Commun..

[7] Rathee D., Rangi S., Chakarvarti S.K., Singh V.R. (2014). Recent Trends in Wireless Body Area Network (WBAN) Research and Cognition Based Adaptive WBAN Architecture for Healthcare. Health Technol..

[8] Le T.T., Moh S. (2016). An Interference-Aware Traffic-Priority-Based Link Scheduling Algorithm for Interference Mitigation in Multiple Wireless Body Area Network. Sensors.

[9] Biglieri E., Goldsmith A., Greenstein L., Mandayam N., Poor H. (2012). Capacity of cognitive radio networks. Principle of Cognitive Radio.

[10] Chávez-Santiago R., Jankunas D., Fomin V.V., Balasingham I. Dual-band Cognitive Radio for Wearable Sensors in Hospitals. Proceedings of the 8th International Symposium on Medical Information and Communication Technology (ISMICT).

[11] Chávez-Santiago R., Balasingham I. Cognitive Radio for Medical Wireless Body Area Networks. Proceedings of the IEEE 16th International Workshop on Computer Aided Modeling and Design of Communication Links and Networks (CAMAD).

[12] Ahmed T., Moullec Y. (2017). A QoS Optimization Approach in Cognitive Body Area Networks for Healthcare Applications. Sensors.

[13] León O., Hernández-Serrano J., Garrigues C., Rifà-Pous H. (2015). A Cognitive-radio-based Method for Improving Availability in Body Sensor Networks. Int. J. Distrib. Sens. Netw..

[14] Chepuri S.P., de Francisco R., Leus G. Performance Evaluation of an IEEE 802.15.4 Cognitive Radio Link in the 2360-2400 MHz Band. Proceedings of the IEEE Wireless Communications and Networking Conference.

[15] Nhan N.Q., Gautier M., Berder O. Asynchronous MAC protocol for spectrum agility in Wireless Body Area Sensor Networks. Proceedings of the International Conference on Cognitive Radio Oriented Wireless Networks and Communications (CROWNCOM).

[16] Bhandari S., Moh S. (2015). A Survey of MAC Protocols for Cognitive Radio Body Area Networks. Sensors.

[17] Le T.T.T., Pan S., Moh S. Link Scheduling for Cognitive Radio Body Area Networks. Proceedings of the 6th International Conference on Smart Media and Applications (SMA 2017).

[18] Cotton S.L., D’Errico R., Oestges C. (2014). A Review of Radio Channel Models for Body Centric Communications. Radio Sci..

[19] Hu Z.H., Nechayev Y., Hall P. Measurements and statistical analysis of the transmission channel between two wireless body area networks at 2.45 GHz and 5.8 GHz. Proceedings of the 2010 Conference Proceedings ICECom, 20th International Conference on Applied Electromagnetics and Communications.

[20] Zurita Ares B., Park P.G., Fischione C., Speranzon A., Johansson K.H. On Power Control for Wireless Sensor Networks: System Model, Middleware Component and Experimental Evaluation. Proceedings of the 2007 European Control Conference (ECC).

[21] Saleem Y., Rehmani M.H. (2014). Primary Radio User Activity Models for Cognitive Radio Networks: A Survey. J. Netw. Comput. Appl..

[22] Shen Q., Liu J., Yu H., Ma Z., Li M., Shen Z., Chen C. Adaptive Cognitive Enhanced Platform for WBAN. Proceedings of the IEEE/CIC International Conference on Communications in China (ICCC).

[23] Salim S., Moh S. (2016). An Energy-efficient Game-theory-based Spectrum Decision Scheme for Cognitive Radio Sensor Networks. Sensors.

[24] Abo-Zahhad M., Farrag M., Ali A., Amin O. An energy consumption model for wireless sensor networks. Proceedings of the 5th International Conference on Energy Aware Computing Systems & Applications.

